# Recent Advance on Mesoporous Silica Nanoparticles-Based Controlled Release System: Intelligent Switches Open up New Horizon

**DOI:** 10.3390/nano5042019

**Published:** 2015-11-25

**Authors:** Ruijuan Sun, Wenqian Wang, Yongqiang Wen, Xueji Zhang

**Affiliations:** Research Center for Bioengineering & Sensing Technology, School of Chemistry and Biological Engineering, University of Science and Technology Beijing, Beijing 100083, China; E-Mails: srj020322@163.com (R.S.); wang_8876@sina.com (W.W.); zhangxueji@ustb.edu.cn (X.Z.)

**Keywords:** controlled release, mesoporous materials, smart materials, stimulus response, nano-switch, free-blockage switch

## Abstract

Mesoporous silica nanoparticle (MSN)-based intelligent transport systems have attracted many researchers’ attention due to the characteristics of uniform pore and particle size distribution, good biocompatibility, high surface area, and versatile functionalization, which have led to their widespread application in diverse areas. In the past two decades, many kinds of smart controlled release systems were prepared with the development of brilliant nano-switches. This article reviews and discusses the advantages of MSN-based controlled release systems. Meanwhile, the switching mechanisms based on different types of stimulus response are systematically analyzed and summarized. Additionally, the application fields of these devices are further discussed. Obviously, the recent evolution of smart nano-switches promoted the upgrading of the controlled release system from the simple “separated” switch to the reversible, multifunctional, complicated logical switches and selective switches. Especially the free-blockage switches, which are based on hydrophobic/hydrophilic conversion, have been proposed and designed in the last two years. The prospects and directions of this research field are also briefly addressed, which could be better used to promote the further development of this field to meet the needs of mankind.

## 1. Introduction

Mesoporous silica nanoparticles (MSNs) have been intensively studied and vastly developed since the first preparation of MCM-41 in 1992 [1]. A great variety of mesoporous silica materials, such as SBA, MSU and FSM, have been developed using different synthetic approaches, including fast self-assembly, soft and hard templating, modified Stöber method, dissolving reconstruction, and modified aerogel approaches [2,3,4,5]. A major goal of this research efforts is to make MSNs possess remarkable advantageous characteristics [6,7,8], such as good biocompatibility, chemical stability, high surface areas, uniform and tailor-made morphologies and pore sizes, as well as versatile functionalization of surfaces and pores. In recent years, MSNs have been investigated for application in controlled drug release systems, attributed to the properties mentioned above. Especially, the development of a responsive MSN-based controlled release system is very important to improve drug efficacy and reduce drug side effects. In addition, MSNs also have been widely used in catalysts, dye-doped imaging and sensing, adsorption, up-converted luminescent devices, detection, and intelligent anticorrosion coating, due to the performance characteristics of MSNs [9].

In clinical therapy, many drugs, especially antitumor drugs such as Taxol, which has cellular toxicity, cause severe damage to normal cells during the medical treatment. To overcome this dam, a widely used strategy is to design a specific, targeted, controlled drug release system that could transport an effective dosage of drug molecules to targeted cells and tissues. As outlined below, several preconditions need to be incorporated into such an efficient controlled drug release system: (1) the drug carrier material should be biocompatible; (2) sufficient dosage of drug can be loaded; (3) zero premature release with no leaking of drug can be achieved [10]; (4) the drug can be delivered to the targeted site and released in a controlled manner [11]; (5) proper release rate can be sustained to achieve effective local drug concentration.

An important prerequisite for designing an efficient controlled release system is the ability to transport the desired guest molecules to the targeted site and release the drug in a controlled manner [11]. The premature release of the drug before reaching the targeted cells or tissues puts forward a challenging problem to all controlled release systems. The release mechanism of many current biodegradable polymer-based controlled drug release systems relied on the hydrolysis-induced erosion of the carrier structure. However, polymer materials are extremely unstable under water, and these systems are barely able to avoid premature releasing. Also, such systems typically employ organic solvents for drug loading, which sometimes could trigger undesirable alterations of the structure and/or function of the encapsulated drug [12]. To overcome the weakness of polymers, MSNs are designed to serve as drug carriers. Hoekstra and Pagano *et al.*, investigated the correlation of particle size and non-phagocytic eukaryotic cells’ internalized particles, and demonstrated that submicron particles could well be internalized by most cells [13,14]. Vallet-Regí *et al.* first used MSNs as a drug carrier, and they also further studied the effective uptake and release characteristics of drugs [15]. It can be noted that the first MSN-based reversible light-responsive controlled release system was constructed through designed molecular switches by Tanaka *et al.* [16]. Since then, MSN-based controlled release systems have become a hot topic in nanotechnology, materials science, clinical medicine, and many other fields.

In this review, we will mainly focus on nano-switches in MSNs. The switches are defined as the entities that can change their shape or location in response to external stimulus. Different responses to external environmental stimuli, including light, pH, redox processes, competitive binding, chemical signals, and biological inputs, are the crucial prerequisites for successfully constructing a controlled drug release system. This paper reviews the different types of nano-switches and their working principles, summarizes the recent developments of different types of MSN-based controlled release systems, and outlines prospects for the direction of future development in this field.

## 2. Non-Functionalized MSNs-Based Controlled Release Systems

Lin *et al*. elucidated the effect of both the pore and particle morphology of MSNs on the performance of controlled drug release systems, and they employed particular room-temperature ionic liquid (RTIL) templated MSNs for the controlled release of antibacterial agents [17]. A series of RTILs containing MSN with various porous structures and particle shapes, such as spheres, ellipsoids, rods, and tubes, were synthesized by using different RTIL templates. By changing the RTIL template, they got the MCM-41 type of hexagonal mesopores, the rotational moiré type of helical channels, and wormhole-like porous structures. This nanodevice delivered antibacterial ionic liquids against *Escherichia coli* K12. The experimental results demonstrated that the antibacterial activity was dependent on the diffusional release rate of the pore-encapsulated RTIL, which was governed by the particle and pore morphology of the MSNs, and spherical with hexagonal RTIL-containing MSNs exhibited a superior antibacterial activity compared with the tubular with wormhole RTIL-containing MSNs. This work demonstrated that the particle and pore morphology of MSNs have a profound influence on controlled release behavior [18].

Han *et al.* employed different concentrations of template to prepare a series of MSNs with controlled microstructural characteristics by the binary surfactant templated synthesis approach [19]. The study also greatly discussed the relationship between the surfactant concentration and the MSNs’ structural development. The *in vitro* drug-releasing kinetics was revealed via loaded Ibuprofen (IBU). The study proved that the length and curvature of the nanopores mainly affected the loaded quantity and the drug release rate. Meanwhile, Zhu *et al.* employed MSNs to study the adsorption and release of bulky biomolecule heparin [20]. Their performance of adsorbing and releasing heparin was assessed with the greatest seriousness, both enlarged pores and organic modifications significantly promoted the adsorption and prolonged the release of heparin in MSNs.

## 3. Functionalized MSN-Based Controlled Release Systems

As mentioned regarding non-functionalized MSN-based controlled release previously, although drug molecules diffused in the MSNs’ nanopores could regulate drug release behavior, this control behavior could only play a role in sustained release, but could not implement blocking drug molecules under normal physiological conditions. As a consequence, some responsive molecules can be modified on the MSNs’ surface or nanopores. Such functionalized MSNs not only block nanopores and prevent drug molecules from releasing prematurely, but they also could respond to external stimuli and release the drug molecules at specific sites. Thus far, researchers have developed a sequence of stimuli-responsive controlled release systems based on MSNs, and the stimuli include light, pH, temperature, competitive binding, enzymes, redox activation, and other signals. Most of these devices have showed excellent behaviors in the controllable release of drugs.

### 3.1. Redox-Responsive Controlled Release Systems

Many human diseases are associated with redox homeostasis in the body. Cancer is one of the major enemies threatening human beings, with some studies demonstrating that liver cancer cells are rich in glutathione, which has a strong reduction performance [21]. In addition, the change of redox homeostasis is also considered to be a leading cause of neurodegenerative disease, atherosclerosis, heart disease, and other cardiovascular diseases or chronic diseases of internal organs. Accordingly, it is of considerable importance to design a redox-responsive controlled release system for the intracellular transport of drugs to kill cancer cells and treat other diseases.

**Figure 1:**
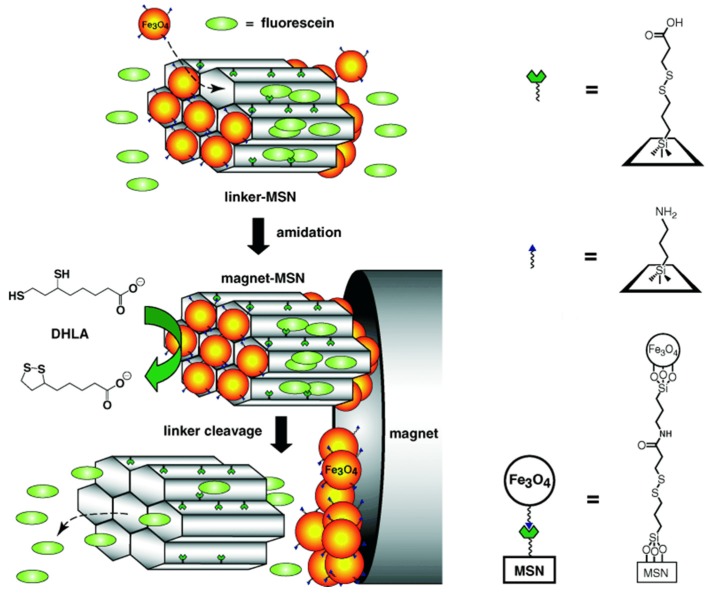
Schematic of the stimuli-responsive controlled release system (magnet-MSN) based on MSNs capped with Fe_3_O_4_ nanoparticles. Reproduced with permission from [23]. Copyright John Wiley and Sons, 2005.

The first example of a redox-responsive MSN-based controlled release system has been constructed using surface-derivatized cadmium sulfide (CdS) nanocrystals as chemically removable caps to trap drug molecules inside the organically functionalized MSN nanopores by Lin and coworkers in 2003 [22]. The nanopores of the drug-loaded MSNs were capped *in situ* by allowing the nanopores’ surface-bound 2-(propyldisulfanyl) ethylamine functional groups to covalently capture the water-soluble mercaptoacetic acid-derivatized CdS nanocrystals. Attesting that this system could block drug molecules for a long time, the premature release of drug molecules was also effectively prevented under normal physiological conditions *in vitro*. However, the resulting disulfide linkages between the MSNs and the CdS nanoparticles were chemically labile in nature, with the presence of various disulfide-reducing agents, such as dithiothreitol (DTT) and mercaptoethanol (ME), and disulfide linkages could be cleaved. This important attempt set off a wave of researching MSN-based controlled release. In 2005, Lin *et al*. further designed a stimuli-responsive controlled release system based on MSNs capped with superparamagnetic iron oxide nanoparticles (Fe_3_O_4_) [23]. As depicted in Figure 1, the disulfide linkages between the MSNs and the Fe_3_O_4_ nanoparticles could be cleaved with disulfide-reducing agents to release the entrapped drugs from the nanopores, successfully implementing redox-responsive controlled release. At the same time, the magnetic motor effect was attractive for the development of site-specific controlled drug release systems, significantly improving the efficiency of intracellular uptake. Thus, this system has met the needs of site-specific drug delivery and controlled release. We envision that a magnet-MSN controlled release system could play a significant role in the development of new generations of site-selective controlled release devices and interactive nano-sensors. This team also loaded the MSNs with the gene and capped the nanopores with gold nanoparticles to keep the gene from leaching out [24]. Following the same design principle, luciferin was loaded in the nanopores of the MSNs and entrapped with disulfide-linked AuNPs. Luciferase was physisorbed on the PEGylated external surface of the Au-MSNs through electrostatic interactions, so the redox-responsive controlled release system was constructed to achieve biocatalytic function in the cell by Lin team in 2011 [25]. The innovation of the system was designed to realize the imaging and quantification of intracellular catalysis, also providing a picturesque route to monitor tumor growth and metastasis by means of bioluminescence and using intracellular ATP and glutathione (GSH) levels as indicators, thus supplying a theoretical basis for researching tumor physiological processes and the treatment of cancer.

**Figure 2:**
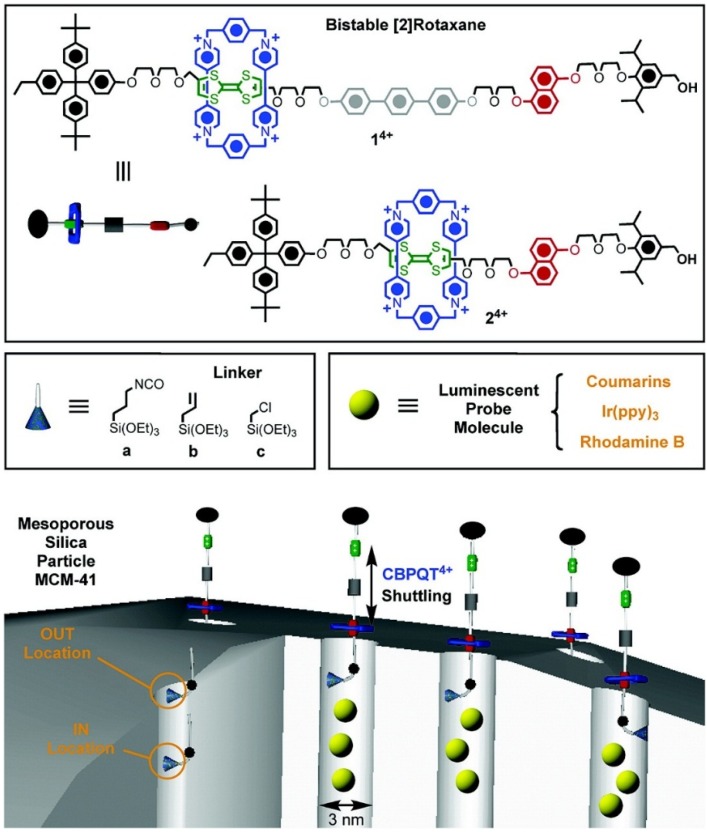
Depiction of the assembly of the components to form nanovalves with the structural formulas of the bistable [2] rotaxanes 1^4+^ and 2^4+^, the three silane linkers **a**, **b**, and **c** used in this study, as well as the graphical representations of luminescent probe molecules and the possible positions (IN and OUT) of the linkers relative to the pore orifice. The pores are loaded when the valves are open and the probe molecules are trapped inside the pores when the valves are closed. The trapped molecules are released when the valves are reopened. The cycle can be repeated over and over again. Reproduced with permission from [28]. Copyright American Chemical Society, 2007.

In 2004, Zink *et al*. first used pseudorotaxane [DNPD⊂CBPQT]^4+^ as a gatekeeper. A gatepost based on a tethered 1,5-dioxynaphthalene (DNP)-containing derivative (DNPD) was installed onto the MSNs’ surface, and cyclobis-(paraquat-*p*-phenylene) (CBPQT^4+^), which recognizes DNP units through a cooperative array of noncovalent interactions, serving as the gate of the nanopores, built a redox-responsive MSN-based controlled release system [26]. The specific signal was an external reducing reagent (NaCNBH_3_) that opened the nanovalve and allowed the release of the luminescent molecules. Subsequently, Zink *et al*. further developed the gatekeeper with pseudorotaxane [DNPD⊂CBPQT]^4+^, and constructed a bistable, reversible redox-responsive nano-switch [27]. The nano-switch was controlled by simple redox chemistry with mild redox reagents and relied on the sliding of rings on the bistable thread without destroying any covalent bonds. Following their previous study, the same group has optimized the pseudorotaxane nano-switch, synthesized from bistable rotaxanes and attached at different positions on the MSNs, and expanded the structure-property relationships of this class of molecular nano-switch, as shown in Figure 2 [28]. It proved that linear molecules were attached at the interiors of the nanopores, and the short silane linker had a better blocking effect; nevertheless, the distance between two binding recognized regions had little effect on the switching performance. These studies were instructive for the future design of pseudorotaxane nano-switch-based controlled release systems, not only redox-responsive systems.

In 2008, Feng *et al*. reported a cross-linked polymeric network, which was used as a “gatekeeper” on the surface of MSNs for the first time. The cross-linked polymeric network worked as a nano-switch based on redox reactions in response to redox signals. Poly(*N*-acryloxysuccinimide) were attached at the pore entrance of MSNs. The nanopores of MSNs were blocked by the addition of cystamine, a disulfide-based bifunctional primary amine, which could react with *N*-oxysuccimide groups along the polymer chain, and in consequence polymer chains be cross-linked [29]. The nanopores of MSNs could be reopened though cleaving the disulfide bond of cystamine in the presence of disulfide-reducing agents such as dithiothreitol (DTT). In 2009, Wang *et al*. reported an efficient cancer cell-targeting and intracellular controlled release system based on aptamer functionalized polyelectrolyte multilayers (PEM)-coated MSNs for cancer therapy [30]. PEM were used to prevent the premature release of drugs during the delivery process and controllably release drugs under reducing conditions, such as intracellular cytosolic space. Moreover, cancer cell-specific aptamers could be used as an effective way to recognize target cancer cells. As a result, the proposed controlled release system based on MSNs-PEM-aptamers provided a foundation for further exploring the controlled release system *in vivo*. Additionally, cyclodextrins were also selected to modify the MSNs via the disulfide linker, which could prevent the premature release of the loaded drugs until the carrier arrived at the cytosolic space [31,32]. In 2012, Li *et al*. introduced a drug controlled release system, where the PEG shell and MSNs were connected via a disulfide bond, MSNs-S-S-mPEG, which is prone to be cleaved upon GSH [33]. The MSNs-S-S-mPEG nanoparticle consists of the MSNs as a container for the drugs, the disulfide bond as a redox-responsive cleavable linker, and the PEG “gatekeeper” that can close or open the nanopores of MSNs. In 2013, the importance of the size of capping agents in stimulus-responsive controlled release systems was studied with poly(propylene imine) dendrimers [34]. Cargo was entrapped with the different sizes of dendrimers anchored on the MSNs through disulfide bonds. The results showed that dendrimers with the diameter of 1.2–1.4 nm were demonstrated to be more effective in drug retention and subsequent release than large-sized dendrimers (>2.4 nm). These findings are significant for optimizing MSN-based controlled release systems as they enable regulating the amount of the released cargo by choosing a capping agent of an appropriate size.

In summary, the redox-responsive MSN-based controlled release systems mainly apply some inorganic nanoparticles, polymers, and biomolecules coupled on the surface of the MSNs via a disulfide bond to block the MSNs’ nanopores, or they employ the pseudorotaxane molecule to reversibly switch the MSNs’ nanopores under redox conditions. The inorganic nanoparticles are not biodegradable, and are easy to enrich *in vivo*. Biomolecules have good biocompatibility and targeting function, so biomolecules serving as nano-switches is the future development direction. In addition, pseudorotaxane molecules as a nano-switch possess reversibility, recyclability, strong operability, and broad application prospects.

### 3.2. pH-Responsive Controlled Release Systems

The pH is one of the most employed internal stimuli to trigger drug release in controlled release systems, because different pathologies such as cancer or inflammation processes exhibit pH changes during their evolution [35,36]. Additionally, when MSNs are internalized inside the cell, the MSNs will be affected by the pH changes in cell, such as in tumor and inflammatory tissues (pH ~ 6.8), endosomes (pH ~ 5.5–6), and lysosomes (pH ~ 4.5–5.0). Therefore, pH-responsive controlled release systems provided a safe and efficient way for controlling drug release in specific sites of the body. Recently, a few successful examples of pH-responsive controlled release systems based on MSNs have been developed.

**Figure 3:**
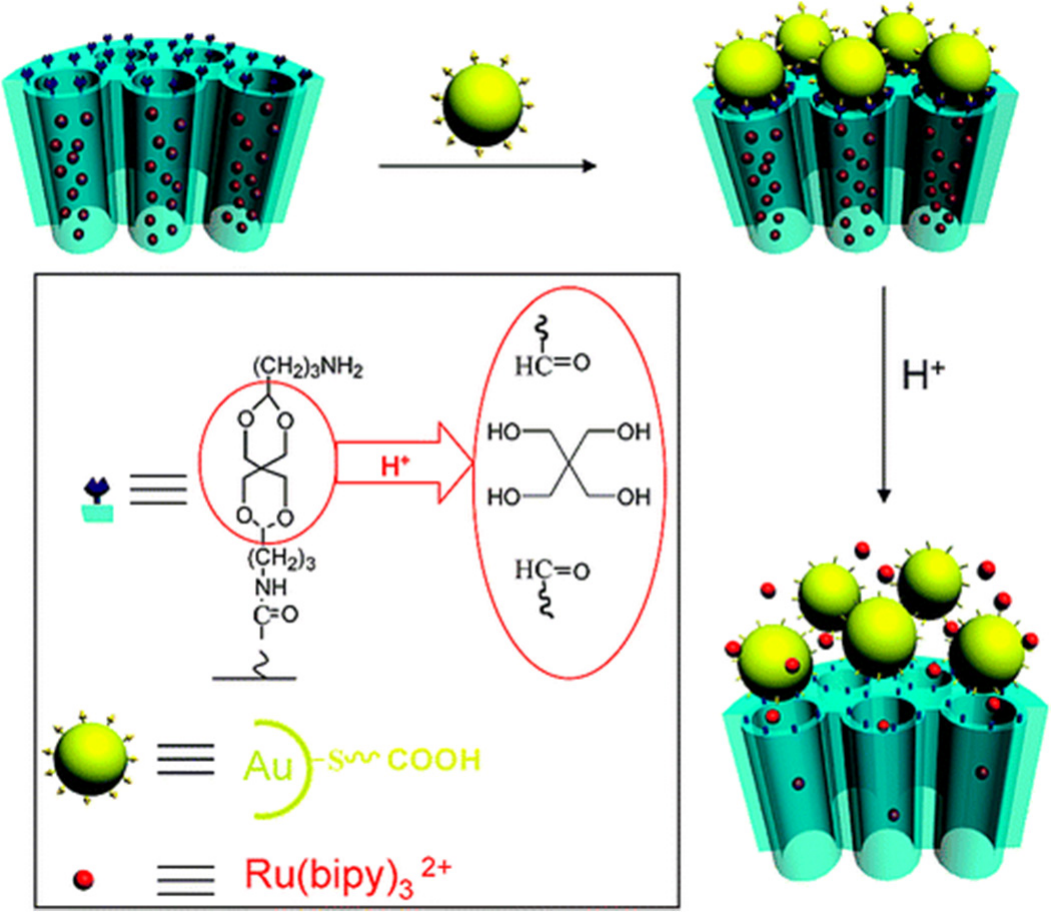
Schematic illustration of pH-responsive nanogated ensemble based on gold-capped MSNs through acid-labile acetal linker. Reproduced with permission from [37]. Copyright American Chemical Society, 2010.

In 2009, Feng and coworkers introduced an acid-cleavable linker into a MSN-based controlled release system and reported a new pH-responsive nanogate ensemble by capping the gold nanoparticle onto the MSNs through an acid-labile acetal linker, as shown in Figure 3 [37]. Carboxylic acid groups were first employed onto the outlet of MSNs (MSN-COOH), the acetal-containing linker was grafted on MSNs by the reaction of excess 3,9-bis(3-aminopropyl)-2,4,8,10-tetraoxaspiro[5.5]undecane with MSN-COOH, followed by the removal of the surfactant template to produce MSN-acetal. The amine groups on the MSN-acetal were then coupled with the carboxylic acid-modified gold nanoparticles (Au-COOH) to cap the gold nanoparticles onto the MSN (MSN-Au). At neutral pH, the linker remained intact and nanopores were blocked with gold nanoparticles to prevent the drug diffusion from the nanopores. At the acidic condition, the hydrolysis of the acetal group would remove the gold nanoparticles and allowed the release of the entrapped drug. Acid-decomposable, luminescent ZnO quantum dots (QDs) have been employed to block the nanopores of MSNs in order to inhibit premature drug release by Zhu [38]. The drug was released from the MSNs nanopores into the cytosol owe to the efficient dissolution of QDs in the acidic environment of cancer cells. Due to the presence of quantum dots, this system could check the cancer evolution, which has great prospects in clinical application.

Cyclodextrins (CDs) have also been a target of concentrated research due to their unique structural, physical, and chemical properties. The most interesting characteristic of CDs was assembled to inclusion complexes with guest molecules. Furthermore, inclusion complexes with various types of guest molecules could be reversibly dissociated by external stimuli [39]. This unique feature has provided a novel route to construct functional pseudorotaxane systems based on CDs as a host. In 2006, Kim *et al*. reported a pH-controlled release of a drug entrapped in the nanopores of MSNs which was blocked by surface-grafted polyethyleneimine (PEI)/CD pseudorotaxane, where CDs play the role of a pH-responsive nano-switches for nanopores of the MSNs [40]. The threading and dethreading process of α-CD onto the PEI block could be controlled by pH variation. In particular, α-CD was threaded onto PEI at pH 11. Furthermore, α-CDs could be dethreaded from the PEI block at pH values below 8, owing to the weak interaction of the protonated PEI chain with the hydrophobic interior of α-CDs; thus, drug was released from the nanopores of MSNs. Based on a similar mechanism, Zink *et al*. reported studies of a simple pH-responsive supramolecular nano-switch based on α-CD/aniline pseudorotaxane structure to control the nanopores opening and closing on the surface of MSNs, and thus controlling the release of the drug [41]. Since then, this same research group has designed two kinds of pH-responsive controlled release systems with β-CD again [42,43]. Both pH-responsive controlled release systems are more functional and more practical, and have successfully implemented intracellular release behavior and regulate cancer cell apoptosis in human differentiated myeloid (THP-1) and squamous carcinoma (KB-31) cell lines. According to the α-CD pseudorotaxane structure, Zink *et al.* utilized the ion-dipole interaction between cucurbit[6]uril (CB[6]) and bisammonium stalks to construct a pH-responsive nano-switch [44]. Six glycouril units strapped together via pairs of bridging methylene groups between nitrogen atoms to form CB[6], which is a pumpkin-shaped polymacrocycle with D_6h_ symmetry [45]. In this study, pseudorotaxanes consisting of bisammonium stalks and CB[6] rings were constructed on the surface of MSNs, and the pH-dependent binding of CB[6] with the bisammonium stalks was employed to control the release of the drug from the nanopores of the MSNs. At neutral and acidic solution, the CB[6] rings encircled the bisammonium stalks strongly to block the nanopores efficiently when employing tethers of suitable lengths. Deprotonation of the stalks upon the addition of base brought about the spontaneous dethreading of the CB[6] rings and the unblocking of the MSNs’ nanopores. However, the pH value to unblock the nanopores was generally about 10, so this controlled release system was unsuitable for biological applications. Thus, the controlled release system has been optimized by Zink *et al.* [46]. Pseudorotaxanes consisting of trisammonium stalks and CB[6] rings were constructed on the surface of MSNs in this new design, and blocked the MSNs’ nanopores at neutral pH and opened the MSNs’ nanopores under weakly acidic conditions and released drugs, raising the possibility for biological applications of such a controlled release system. In 2013, Fu and coworkers reported acid and base dual-responsive cucurbit[7]uril pseudorotaxanes, which have been immobilized on the surface of MSNs as the supramolecular nano-switch to control the release of a drug in response to widespread pH changes. This system could completely entrap the drug at a neutral pH, and release a drug accurately and timely either under acidic or alkaline conditions [47].

In 2005, Xiao *et al*. utilized an oppositely charged ionic interaction between carboxylic acid-modified SBA-15 silica rods and polyelectrolyte, and constructed an efficient pH-responsive controlled release system [48]. The controlled release system consisted of carboxylic acid-modified SBA-15 mesoporous silica rods and poly-(dimethyldiallylammonium chloride) (PDDA) as a container, and released the drug from nanopores. At pH 6.5, due to a strong interaction between the negative group of COO^−^ and the positive group of PDDA, the state of the gates around the nanopores was almost closed. When the pH value was decreased to 2.0, there was no negative charge on the carboxylic acid-modified SBA-15 silica rods. Therefore, PDDA with a positive charge would be separated from the surface of SBA-15 and then the state of the gates around the nanopores was completely opened. Subsequently, a series of pH-responsive controlled release systems with polymers were constructed by scientists with polyacrylic acid [49,50,51], poly(4-vinylpyridine) [52], chitosan [53], diblock polymer(poly(PDM-b-PEGMA)) [54], drugs/surfactant micelles-co-loaded MSNs [55], copolymer of poly(allylamine hydrochloride)/poly(styrene sulfonate) [56] and poly(*N*,*N*-dimethylaminoethyl methacrylate) [57].

In summary, compared with the redox-responsive controlled release system, the pH-responsive controlled release system based on MSNs is an intracellular responsive release system, and it also has wider application and better system effectivity. Up until now, some of the pH-responsive controlled release systems worked at low pH (pH 3~4), much lower than the acidic conditions inside the cancer cells, and such a system cannot be applied for intracellular controlled release. However, the pH-controllable switch of most polymer electrolytes does not have this problem, and their switch condition is about pH 5 to 6, in line with the cell’s weakly acidic conditions.

### 3.3. Light-Responsive Controlled Release Systems

All the above-mentioned controlled release systems were designed based on the intracellular environment, which could release a drug in a specific cell and corresponding condition. In order to improve the operability of the controlled release system, new external stimuli-responsive controlled release systems have become a hot point. Light is an important stimulus signal, among others. Using light to trigger the drug release has many advantages, including remote responsiveness, non-invasiveness, highly controllable properties, low toxicity, and convenient operation.

The first light-responsive controlled release system was constructed with coumarin-modified MSNs. Tanaka and coworkers revealed the regulation of loaded, retention, and controlled release of drug through the photo-controlled and reversible intermolecular dimerization of coumarin derivatives attached to the nanopores of MSNs [16]. By exposure to light greater than 310 nm, the resulting photodimerization of the coumarin led to the isolation of nanopores through blocking the nanopores’ entrance with cyclobutane dimers. The drug-loaded MSNs were then exposed to 250 nm UV light to cleave the cyclobutane rings of the coumarin dimers and make the subsequent release of the stored drug. In 2012, a reversible light-responsive system was designed based on thymine-derivative functionalized MSNs [58]. The nano-switch was developed based on the photodimerization-cleavage cycle of thymine upon different irradiation. In the system, thymine derivatives were grafted on the nanopore outlets of MSNs. The irradiation of 365 nm UV light resulted in the formation of a cyclobutane dimer in the nanopores’ outlets, subsequently leading to the blockage of the nanopores and strongly inhibiting the diffusion of the drug from the nanopores. With 240 nm UV light irradiation, the photocleavage of the cyclobutane dimer opened the nanopores and allowed the release of the entrapped drug.

**Figure 4:**
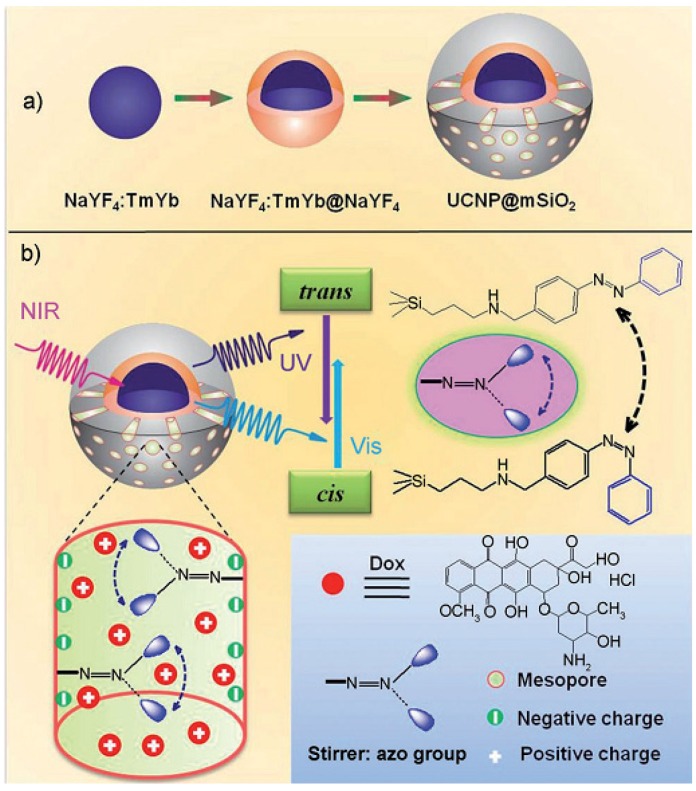
(**a**) Synthetic procedure for up-converting nanoparticles coated with a MSN outer layer. (**b**) The schematic of NIR light-triggered doxorubicin release by making use of the up-conversion property of UCNPs and *trans-cis* photoisomerization of azobenzene group molecules grafted on MSNs. Reproduced with permission from [62]. Copyright John Wiley and Sons, 2013.

In 2008, Zink *et al*. reported the use of azobenzene (AB) derivatives as gatekeepers in and on MSNs, and drugs were expelled from the MSNs under photocontrol [59,60,61]. The most representative controlled release system among them was designed by β-CD and AB; AB derivative-modified MSNs are capped with pyrene-substituent β-CD (Py-β-CD), which was designed as a light-operated nano-switch. Azobenzene undergoes isomerization upon UV light irradiation at certain wavelengths. β-CD possesses a high binding affinity with the *trans*-azobenzene derivative, but barely interacts with its *cis*-configuration. Therefore, the dissociation of β-CD from the azobenzene stalk was induced by the conformational change of azobenzene from *trans* to *cis* upon irradiating with 351 nm UV light. Thus, the release of the drug from MSNs was realized. In 2013, Shi and coworkers reported a novel strategy of near-infrared (NIR) light-responsive anticancer drug release based on MSNs coated up with a converting nanoparticle (UCNP) structure, designated as UCNP@MSNs and shown in Figure 4 [62]. By coating NaYF_4_:Tm, Yb@NaYF_4_ with azobenzene group-modified MSNs and using 980 nm light, the amount of the released drug could be fully controlled through varying the intensity and/or time duration of the NIR light irradiation.

In 2009, Lin and coworkers reported on the synthesis of AuNP-capped MSNs for the light-responsive intracellular controlled release of an anticancer drug [63]. Firstly, a light-responsive linker (thioundecyl-tetraethyleneglycolester-*o*-nitrobenzylethyldimethyl ammonium bromide, TUNA) was modified onto the surface of the AuNPs, which were positively charged in phosphate buffer solution (PBS, pH 7.4). The capping mechanism of the AuNP-MSNs system relied on the electrostatic interaction between the positively charged AuNPs and the negatively charged MSNs in water. The photolabile linker covalently attached to the surface of AuNPs would be cleaved through photo-irradiation, resulting in the formation of the cationic compound as well as the negatively charged, thioundecyltetraethyleneglycolcarboxylate (TUEC)-functionalized AuNPs (NC-AuNPs). The charge repulsion between the NC-AuNPs and MSNs would then open nanopores and induce the release of the drug. In the same year, Kim *et al*. constructed an analogous light-responsive controlled release system with cyclodextrin (CD)-covered MSNs [64]. Upon exposure to UV light, the drug was released from the nanopores of MSNs after the removal of the CD “gatekeeper”, which was linked on the surface of MSNs through a photocleavable *o*-nitrobenzyl ester moiety. Lin and coworkers employed 2-nitro-5-mercaptobenzyl alcohol-functionalized CdS nanoparticle-blocking nanopores through a photocleavable carbamate linkage. Drug release from this controlled release system was also triggered upon irradiation with UV light [65].

Light-responsive controlled release systems have many advantages, including remote operation, and easily controllable properties in arbitrary positions or times, thus light has been widely applied in the field of controlled drug release systems. Additionally, due to light-responsiveness, controlled release systems have been designed to respond to most of the ultraviolet light signals, and it is difficult to apply to this the cells and *in vivo* compared to near-infrared and far-infrared light, which can penetrate tissue and cause no damage to cells and tissues; hence, the near-infrared- and far-infrared-responsive controlled release systems are the future development direction in this field.

### 3.4. Temperature-Responsive Controlled Release Systems

Tumors, inflammation, or infection processes can cause moderate temperature increases of up to 4–5 °C. Grafting a temperature-sensitive nano-switch on the surface of MSNs makes it possible to design a temperature-responsive controlled release system. The most common temperature-sensitive polymers are based on poly-*N*-isopropylacrylamide (PNIPAM) and its derivatives. Below a lower critical solution temperature (LCST), these polymers exhibit a hydrophilic extended state, which creates a diffusion bottleneck that hampers the drug release. When the temperature is higher than the LCST, the water is excluded from these polymers, which collapse to release the loaded drug. PNIPAM was modified on the internal surface of the MSNs through atom transfer radical polymerization (ATRP) by Lopez and coworkers, who confirmed that the PNIPAM-functionalized MSNs can release drugs at a high temperature (50 °C), and inhibit the release of drugs at a low temperature (25 °C). Based on this phenomenon, the team constructed a temperature-responsive controlled release system [66]. Further work has been done to preformed thiol-functionalized MSNs with pyridyl disulfide-terminated poly(*N*-isopropylacrylamide) (PNIPAM-S-S-Py) by Oupický *et al*. [67]. The polymer was in the random coil conformation at room temperature (below the LCST of the polymer), which resulted in the release of loaded molecules. However. a low level of the drug leaked out due to globule conformation of the polymer at 38 °C (above LCST). In this system, PNIPAM was grafted on the external surface of the MSNs and, along with a greater-than-10-fold improvement in drug retention in the “nanopore-closed” conformation, was opposite to the system in Lopez’s work. Additionally, better biocompatible polymers such as sulfobetaine copolymers [68] poly(ethyleneoxideb-*N*-vinylcaprolactam) [69], and orcopolymer-lipid bilayers [70] have been reported.

### 3.5. Magnet-Responsive Controlled Release Systems

**Figure 5:**
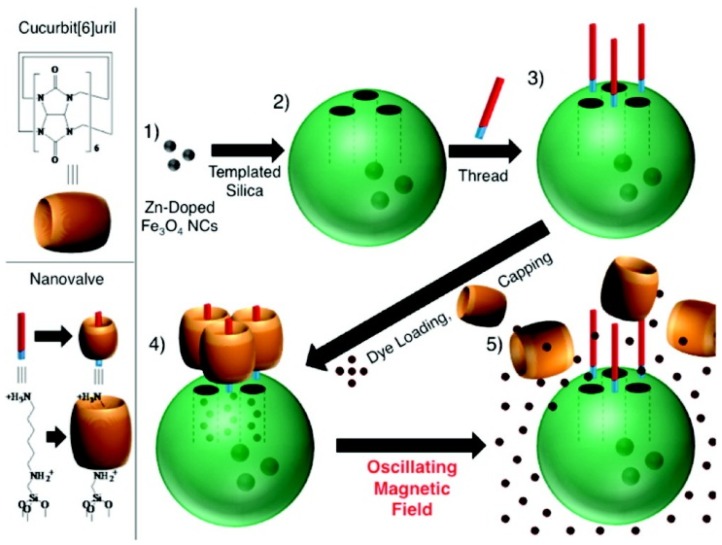
Schematic illustration of the synthesis and operation of a magnet-responsive controlled release system, using ZnNCs encapsulated within MSNs. Reproduced with permission from [72]. Copyright American Chemical Society, 2010.

The magnet-responsive controlled release system is related to the temperature case to a large extent. The encapsulation of magnetic particles within the MSNs can generate thermal energy under an externally applied alternative magnetic field. In 2010, Chen *et al*. showed a novel nanocarrier (MSN@Fe_3_O_4_), which was constructed by capping MSNs with monodispersed Fe_3_O_4_ nanoparticles through chemical bonding. Amine-MSN nanopores were covalently capped through amidation of the 3-aminopropyltrimethoxy silane bound at the nanopores’ surface with *meso*-2,3-dimercaptosuccinic acid-functionalized superparamagnetic Fe_3_O_4_ nanoparticles (DMSA-Fe_3_O_4_ NPs) [71]. Without a magnetic stimulus, few drugs could be released from the MSN@Fe_3_O_4_. Fe_3_O_4_ nanoparticles could be removed from the surfaces of the MSNs caused by the breaking of chemical bonds under magnetic stimulus, which subsequently resulted in fast, responsive drug release. Magnetic nanocrystals have the characteristic of hyperthermic effects when placed in an oscillating magnetic field. Zink *et al*. prepared zinc-doped iron oxide nanocrystals (ZnNCs) containing MSNs, which were surface-modified with cucurbit[6]uril pseudorotaxanes, as shown in Figure 5 [72]. The system was simple to disassemble and easily released the drug when the ZnNCs generated local internal heating upon the application of an alternative magnetic field. This system promised to be a noninvasive, externally controlled drug release system to use for cancer therapy. In 2011, Vallet-Regí *et al*. designed a smart controlled release system through DNA/magnetic nanoparticle conjugates, which were capable of capping the nanopores of the complementary strand-functionalized MSNs due to DNA hybridization [73]. The assembly and disassembly of the DNA led to a reversible release mechanism. Moreover, the magnetic component of the whole system allowed reaching hyperthermic temperatures (42–47 °C) under an alternating magnetic field, and this feature provided the possibility of a remotely magnet-responsive controlled drug release.

### 3.6. Biomolecule-Related Controlled Release Systems

Biomolecule-related controlled release systems have been greatly developed in response to the special environmental changes of cells. The incorporation of MSNs and biomolecule-related switches has attracted much attention for their better biocompatibility, accurate responsibility, and milder controlled release conditions when compared to other controlled release systems, which also provides the way to construct MSN-based controlled release systems with the higher specificity and efficiency.

#### 3.6.1. Enzyme-Responsive Controlled Release Systems

Enzyme-responsive controlled release systems have made a pioneering exploration for designing a better biocompatible and intelligent controlled release system. Meanwhile, enzyme-responsive controlled release systems have significant potential in clinical application because some diseases are caused by the overexpression of certain enzymes.

In 2008, Zink *et al*. reported a novel enzyme-responsive controlled release system. Firstly, the MSNs were modified with aminopropyltriethoxysilane to achieve an amine-modified surface, and then alkylated with a tri-(ethylene glycol) monoazide monotosylate unit to give an azide-terminated surface, the α-CD thread onto the tri-(ethylene glycol) chains, effectively blocking the nanopores [74]. In this system, porcine liver esterase appeared to catalyze the hydrolysis of the adamantyl ester stopper, resulting in the dethreading of the α-CD, and the release of the drug from the nanopores. Bein and coworkers employed biotin-avidin as a protease-responsive cap, which was capable of specifically recognizing the binding form of a certain three-dimensional structure, and could effectively block MSNs nanopores [75]. Under the effect of protease, the biotin-avidin complex could be degraded, and it subsequently opened the MSNs’ nanopores to release drug. Therefore, a MSN-based enzyme-responsive controlled release system has been achieved. Kim and coworkers reported an α-amylase and lipase-responsive controlled release system, with α-CD nano-switch [76]. The same year, Amorós *et al*. reported a β-d-galactosidase-responsive controlled release system, where a lactose molecule serves as a nano-switch [77]. Bhatia *et al*. reported the preparation of protease-responsive polymer-coated MSNs and the loading of drugs into both the core and shell of MSNs [78].

#### 3.6.2. Glucose-Responsive Controlled Release Systems

**Figure 6:**
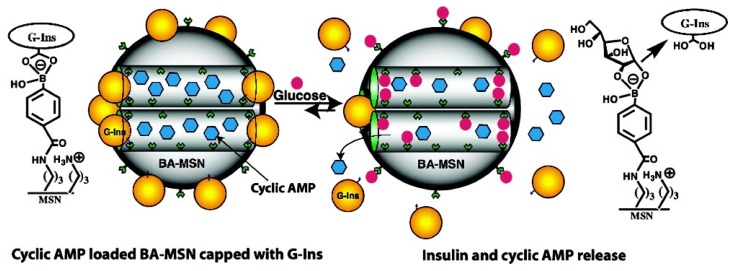
Schematic representation of the glucose-responsive MSN-based delivery system for controlled release of bioactive G-Ins and cAMP. Reproduced with permission from [79]. Copyright American Chemical Society, 2009.

Lin *et al*. have developed a glucose-responsive controlled release system, and successfully demonstrated that phenylboronic acid-functionalized MSNs can serve as an efficient co-delivery system for the controlled release of insulin and cyclic adenosine monophosphate (cAMP) [79]. As depicted in Figure 6, gluconic acid-modified insulin (G-Ins) proteins were immobilized on the surface of MSNs and also served as caps to encapsulate cAMP molecules inside the nanopores of MSNs. The release of both G-Ins and cAMP from the MSNs could be triggered by the presence of saccharides, such as glucose. In 2012, a glucose-responsive controlled release system was described by Lu *et al*. This system was designed based on the competitive combination among glucose oxidase, glucosamine, and glucose [80]. Great controlled release capabilities and high selectivity for glucose were achieved over other monosaccharides.

#### 3.6.3. DNA-Based Controlled Release Systems

**Figure 7:**
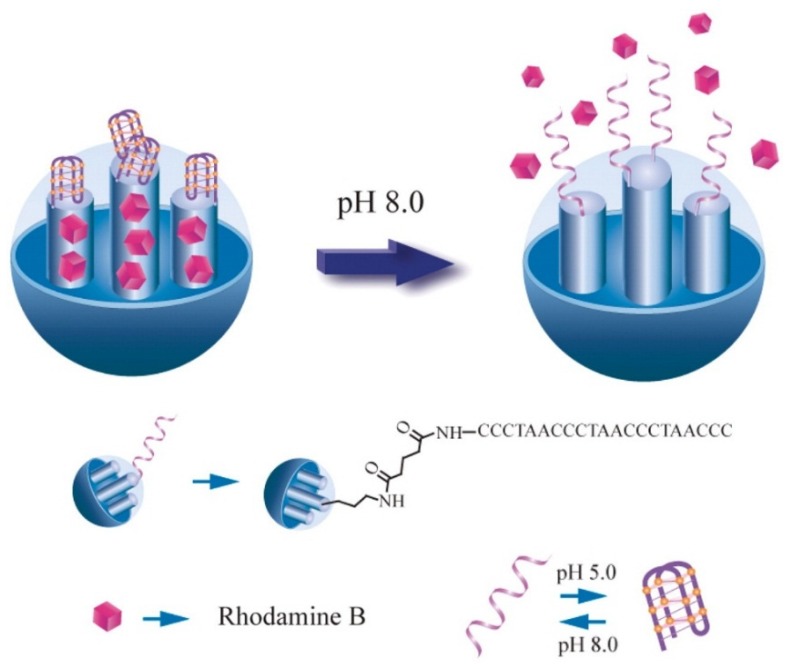
Schematic representation of proton-fueled release of a drug from the pores of MSNs capped with *i*-motif DNA. Reproduced with permission from [84]. Copyright Oxford University Press, 2011.

Over the past two decades, DNA has been recognized as an attractive building block for nanotechnology and materials science due to its conformational polymorphism, programmable sequence-specific recognition, and robust physicochemical nature; these features make it attract a lot of attention in the field of nano-switches. In recent years, DNA-based controlled release systems have become a hot point in the field of controlled release systems [81,82,83,84,85,86,87,88]. Amorós *et al*. reported that the outer surfaces of MSNs were functionalized with 3-aminopropyltriethoxysilane and capped with a single-stranded oligonucleotide. At neutral pH, aminopropyl groups were partially charged and would interact with negatively charged oligonucleotides, resulting in the closing of the nanopores [81]. In the presence of a target complementary strand, it resulted in the hybridization of the two oligonucleotides. Then, the nanopores were uncapped through a highly effective displacement reaction to release the entrapped cargo. In 2010, Qu *et al*. reported a kind of DNA-based controlled release system which opened nanopores and released the drug by relying on a denaturing of duplex DNA by heating or endonuclease hydrolyzation. The principle of this system came from the unique structural motif and self-recognition properties of duplex DNA, including temperature-dependent assembly, as well as the enzymatic recognition of specific encoded bases [82]. In 2011, a novel proton-fueled nano-switch controlled release system was constructed using *i*-motif quadruplex DNA as a cap by the same research group. The *i*-motif DNA cap could smartly respond to a pH stimulus due to simple conformation changes. At a low pH (pH 5.0), the DNA folded into the *i*-motif quadruplex structure, the nanopores were capped by the quadruplex, and the release of the cargo was strongly inhibited. When the pH was increased to basic (pH 8.0), the DNA unfolded to a single-stranded form, the MSN nanopores were spontaneously unblocked, and this resulted in the fast release of the cargo from the nanopores into the aqueous solution, as shown in Figure 7 [84].

**Figure 8:**
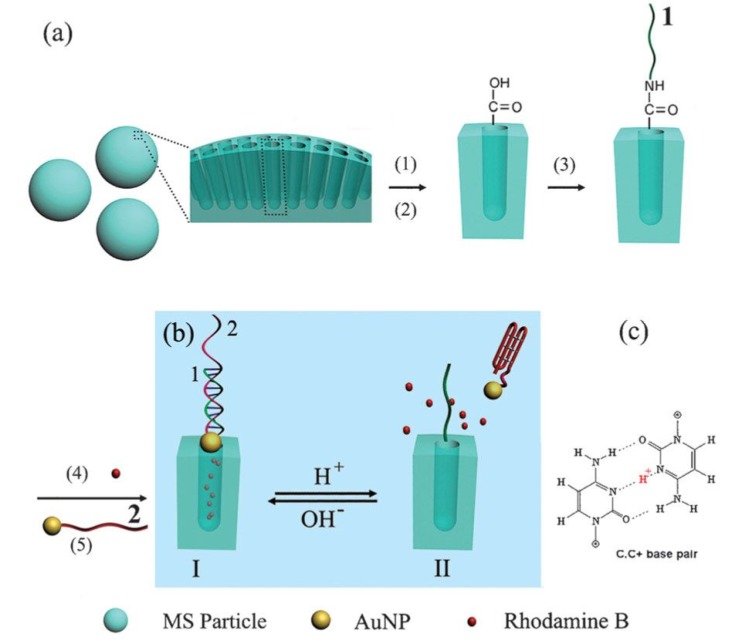
(**a**) Scheme of preparation of DNA-modified MSNs. (1) 3-Aminopropyltriethoxy silane; (2) succinic anhydride and triethylamine; (3) *N*-hydroxy-succinimide (NHS), 1-ethyl-3-(3-dimethylaminopropyl) carbodiimide-HCl (EDC), and NH_2_-ended DNA strand 1; (4) cargo molecules, Rodamine B; (5) DNA 2-functionalized AuNPs. (**b**) The controlled release was modulated by the motor DNA’s conformation change which was driven by changing the pH value of the solution. (**c**) A schematic sketch of the hydrogen bonding between the protonated cytosines. Reproduced with permission from [85]. Copyright The Royal Society of Chemistry, 2011.

A lot of studies about DNA-based controlled release systems have been done by our group. In 2012, a smart pH-responsive controlled release system was designed which was based on the DNA nano-switch controlled organization of AuNPs attached to MSNs, as shown in Figure 8 [85]. In this system, the pH transition controlled the hybridization and dehybridization of DNA between strands 1 and 2. In this design, we prepared the DNA 1-derivatized MSN (MSN-DNA_1_) and the DNA 2-modified AuNPs (Au-DNA_2_), and the DNA 2 exhibited an extended random conformation under neutral to alkaline pH which could further hybridize with the partially complementary strand 1 to form a rigid double-helical form; then AuNPs-DNA_2_ were capped on the nanopores. Nevertheless, in mildly acidic conditions, DNA 2 switched to a folded quadruplex *i*-motif conformation leading to the dehybridization of the duplex and the opening of the nanopores. The pH transition could thus be used to control the close/open of the nanopores of the MSNs through the duplex–quadruplex interconversion of this DNA nano-switch under physiological conditions. This research made a further step toward the development of a more highly efficient controlled release system and novel applications of DNA nanotechnology. In addition, this acid-sensitive release system was highly desired in the treatment of acidic targets, such as tumors and inflammatory tissues. Subsequently, our group designed an intelligent photo-switchable single-molecule nano-switch with a DNA hairpin-loop structure by the incorporation of azobenzene groups in the DNA sequences, which were attached onto the surfaces of the MSNs. Based on the photo-induced conformational transformation of azobenzene, a high-efficiency controlled release system was realized [86]. In 2013, Qu and coworkers also described a targeted intracellular light-responsive controlled release system using photosensitizer-incorporated G-quadruplex DNA-capped MSNs [87]. Bimolecular G-quadruplex DNA ((G_4_T_4_G_4_)_2_) was anchored to the outlet of the MSNs, and served as a cap to encapsulate the drug within the nanopores. Additionally, *meso*-Tetra(*N*-methyl-4-pyridyl)porphine tetra tosylate (TMPyP_4_), a kind of photosensitizer, tended to bind to the G-quadruplex structures. In this way, the quadruplex DNA moiety of the capping could be cleaved via the photosensitized production of reactive oxygen species (ROS) by the incorporated TMPyP4, thus opening the nano-switch of the nanopores and releasing the drug. Target-directing molecules have been immobilized to the external surface of the MSNs to increase the specificity toward cancer cells and minimize the toxicity to the nearby normal tissues.

Aptamers are a kind of specific DNA fragment that can specifically bind various biological molecules (such as enzymes, growth factors, antibodies, gene regulatory factors, cell adhesion molecules, pathogens, *etc.*), and they opened up new a interspace for the design of specific DNA-based nano-switches. Recently, our group and other research groups explored aptamer-responsive MSN-based controlled release systems and obtained excellent controlled release efficiency [88,89]. Our group preformed AuNPs to cap the nanopores with programmable DNA as the linker that could respond exclusively to adenosine [89]. Also, DNA assemblies that could perform “OR” and “AND” logic gate operations were designed where multiple responsibilities were integrated into a single functional device. In our group design of the “OR” gate, potassium ion or thermal-responsive DNA assemblies were designed, and the openings of the nanopores at the MSNs’ surfaces were capped with the AuNPs, which were modified by a potassium ion aptamer sequence. This “OR” logic gate device could respond to two different signals. The “OR” logic gate device had response at least one of the signals was true. Then the adenosine aptamer modified on the AuNPs and cocaine aptamer modified on the MSNs constituted “AND” logic gate device. The “AND” logic gate device had response only when both of the signals were true [90]. Such efficient computation systems could be expected to realize more precise and efficient target release in complicated situations, which represents an important step towards other more practical applications.

#### 3.6.4. Targeted Delivery Controlled Release Systems

The construction of stimuli-responsive controlled release systems for targeted drug delivery to specific cells is of considerable magnitude for the development of clinical medicine. Various components have been employed to block the MSNs for controlled drug release systems, such as pseudorotaxanes, inorganic AuNPs, Fe_3_O_4_, CdS, β-CD. Nevertheless, none of these reports combined blocking MSNs and cell-specific targeting for stimuli-responsive controlled drug release systems.

In 2011, a MSN-based controlled release system, which was blocked with collagen, had great potential for both cell-specific targeting and redox responsiveness [91]. The surface of the MSNs was functionalized with 3-aminopropyltriethoxysilane to result in NH_2_-MSN, which was then reacted with succinic anhydride to produce COOH-MSN. Later on, the conjugate between the disulfide bond linker and the MSN (linker-MSN) was prepared by use of cystamine. Fluorescein isothiocyanate (FITC) was utilized as both a model drug and site marker for intracellular tracing of the MSNs. The linker-MSN/FITC was further covalently coupled with collagen to attach the FITC-loaded nanopores to the MSNs. Finally, a cell-specific targeting moiety, LA-Col-linker-MSN, was produced by grafting lactobionic acid (LA) to the collagen-capped MSNs. Through this series of reactions, Cai has successfully obtained that collagen-capped MSNs could serve as a redox-responsive container for efficient, targeted controlled drug release to cancer cells. More importantly, this system has great potential for future biomedical applications that require *in vivo* controlled, targeted drug release, such as the clinical therapy for liver cancer. Wang and coworkers have designed a novel and general biomolecule-related controlled release system using MSNs based on aptamer target interactions [92]. In this system, AuNPs were modified with ATP aptamer to cap the nanopores of the MSNs. In the presence of ATP molecules, the AuNPs were uncapped due to a competitive displacement reaction, then the drug was released rapidly. In 2014, Zhu *et al*. reported the design of a dual-targeted drug delivery system that was based on DNA-hybrid-capped MSN-coated quantum dots (MSQDs), where the drug release was controlled by miRNA [93]. They synthesized a DNA hybrid, which is composed of an aptamer and an antisense oligonucleotide of miR-21. Aptamer-targeted delivery and miRNA-controlled release were achieved contemporaneously, which resulted in maximum therapeutic efficacy and minimal side effects due to the overexpression of nucleolin and miR-21 in cancer cells. More importantly, this miRNA-responsive controlled drug release system provided a foundation for combining chemotherapy and gene therapy to obtain an optimized therapeutic efficacy in cancer treatment, given the complexity of the regulation network, which was composed of miRNAs and their multiple gene targets. All these researches indicated that the aptamer-target interaction could provide a promising direction for the design of a novel controlled release system, which is specifically governed by target biomolecules. Aptamer-based controlled release systems have a wide range of applications, and that is because aptamers have been obtained for a wide range of targets, including some cancer biomarkers. In summary, the biomolecule-based controlled release system has become the research hot spot for many researchers, due to its excellent biocompatibility, specificity and accurate signal response, and broad prospects in *in vivo* biological applications. Among them, the DNA-based controlled release system is one of the research hot spots. Compared with other biomolecule switches, the DNA-based nano-switch has many advantages, such as high biocompatibility, a simple and varied design and synthesis method, an especially accurate and fast response in a complex environment, and strong anti-interference ability. It occupies an important position in the future design of controlled release systems and in building nanorobots, nano-logic gates and other fields.

### 3.7. Multiple-Responsive Controlled Release Systems

In addition to the single-responsive controlled release systems, researchers have also designed a series of controlled release systems which could respond to complicated environmental stimulation. These multiple-responsive systems have many potential applications for fabricating logic gate operations, and further designing intelligent controlled release systems.

#### 3.7.1. pH- and Cation-Responsive Controlled Release Systems

**Figure 9:**
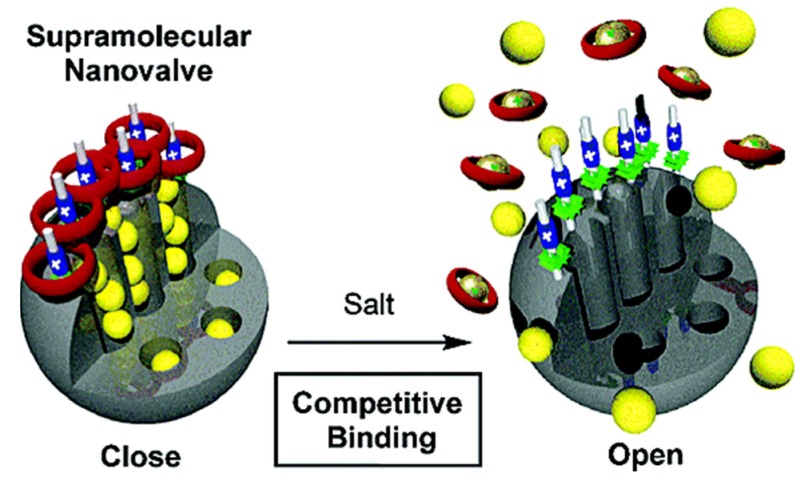
Graphical representation of operating supramolecular nanovalves from DB24C8/dialkylammonium-tethered porous silica particle MSNs. Reproduced with permission from [94]. Copyright American Chemical Society, 2006.

In 2008, Zink and coworkers reported a multiple-responsive controlled release system that was prepared by using dibenzo-[24]crown-8(DB24C8)/dialkylammonium ion pseudorotaxane as the moving parts of nanovalves [94]. In this system, the closed configuration was constituted by the hydrogen bonding between the dialkylammonium ion (tethered at the nanopores orifices) and the DB24C8 rings; thus, the release of the drug from the MSNs was prevented effectively. With the addition of a base (such as triethylamine), the charge of the dialkylammonium moieties was neutralized, and the hydrogen bonding between the dialkylammonium ion and the DB24C8 rings disappeared, leading to the dissociation of the rings and the opening of the nanovalves. This system also offered a selective method to open the nanovalves based on the competitive binding of the DB24C8 ring by complexing agent-metal/fluorodialkylammonium cations. Through trapping the dissociated DB24C8 rings and preventing them from recomplexing with the stalks, this competitive binding activation could vary the equilibrium of the complexation-decomplexation process at the nanopore openings. Therefore, the nanovalves could be opened to release the drug by either pH stimulation with bases or competitive binding with metal/fluorodialkylammonium cations, as shown in Figure 9.

#### 3.7.2. pH- and Anion-Responsive Controlled Release Systems

Eliseo Ruiz *et al*. reported a pH- and anion-responsive supramolecular nano-switch, which was obtained by anchoring suitable polyamines on the nanopore outlets of MSNs [95]. A pH-responsive open/close mechanism arises from the hydrogen-bonding interaction between unprotonated amines, which display poor coverage of the nanopores at neutral pH (fully open gate) and coulombic repulsions between closely located polyammoniums at the nanopore openings (closed gate) at acidic pH. Apart from the pH-responsive mode, the opening/closing of the nano-switch could also be modulated by an anion-responsive mechanism. The interactions between polyamines and a range of anions with different structural dimensions and charges could result in different gates. Depending on the pH, the selected anion could result in a different gate behavior, ranging from ultimately no action (chloride) to complete (ATP) or partial nanopore blockage.

#### 3.7.3. pH- and Light-Responsive Controlled Release Systems

In 2008, Guillem *et al*. proposed a pH- and light-responsive controlled release system where a saccharide derivative was functionalized onto the nanopore outlets of MSNs and was capable of interacting with boronic acid-functionalized AuNPs acting as blockages via the reversible formation of the corresponding boroester bonds [96]. The developed system could be triggered by two simple external stimuli such as pH changes or light in aqueous solution. The hydrolysis of the boroester bond occurs at pH 3, which could lead to the rapid release of the drug from the MSNs’ nanopores. However, the nanopores could be capped with AuNPs and the release is strongly inhibited at pH 5. As a stimulus, when the suitable light was used to release procedures, the AuNPs’ capacity for raising their temperature locally by the absorption of laser light could result in the cleavage of the boronic ester linkage, allowing the release of the entrapped drugs.

Zink’s research group also did some research on multiple-responsive controlled release system [97]. They proposed a dual-responsive controlled release system in which two different types of machines, namely the base-responsive nanovalves based on CB[6]/bisalkylammonium[2]pseudorotaxanes, were tethered to the outer surfaces of MSNs and the photocontrolled nanoimpellers based on azobenzene derivatives were attached to the nanopore walls. The dual-responsive controlled release system reported here relied on light and pH inputs and realized an “AND” logic gate function.

**Figure 10:**
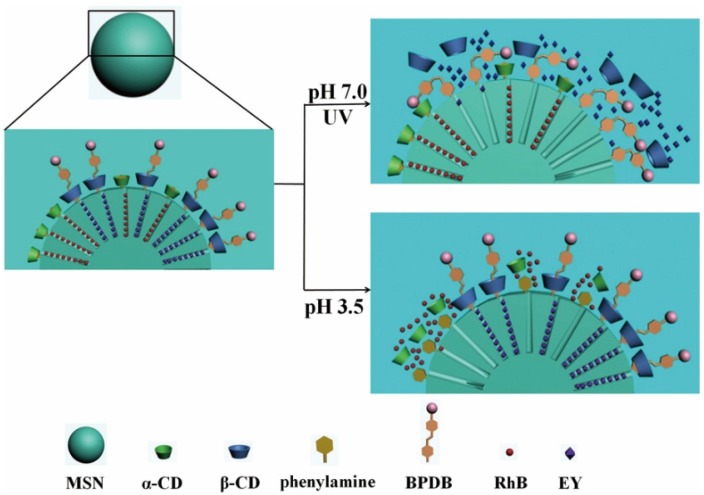
The release process of the dual-dye-loaded MSNs. The dual dyes were loaded into the MSNs separately by pH-controlled nanogates and UV-controlled nanovalves. This system can selectively release Eosin Yellowish (EY) upon UV irradiation (at pH 7.0) and Rhodamine B (RhB) at pH 3.5. Reproduced with permission from [98]. Copyright John Wiley and Sons, 2014.

In 2014, our group demonstrated a selective controlled release system with dual-drug-loaded MSNs. This system could selectively release different kinds of drugs separately when stimulated by different signals (light or pH), as shown in Figure 10 [98].

A supramolecular pseudorotaxane system was composed of α-CD rings on *N*-phenylaminopropyltrimethoxysilane (PhAPTMS) containing a phenylamine (Ph) group. The Ph/α-CD could act as “gatekeepers” of the nanopores on the MSNs due to the strong binding propensity between α-CD and the Ph group. At pH 3.5, Ph/α-CD could be dissociated to unblock the nanopores of the MSNs to trigger the release of the drug. This result was contributed to the much weaker interaction between α-CD and the protonated Ph group. In addition, another group of pseudorotaxanes was assembled by β-CD rings threading onto (*E*)-4-((4-(Benzylcarbamoyl) phenyl)diazenyl) benzoic acid (BPDB) stalks containing an azobenzene group. BPDB/β-CD could act as “gatekeepers” of the nanopores on the MSNs. At pH 7.0, the BPDB/β-CD pseudorotaxanes could be disassociated to open the nanopores to release another drug due to the *trans*-*cis* photoisomerization of the azobenzene unit under UV light. This controlled release system would be an important evolution to develop more effective and complicated nanomedicine and nanodevices for the selective release of multiple drugs under different stimulations.

#### 3.7.4. pH- and Temperature-Responsive Controlled Release Systems

In 2013, a temperature and pH dual-responsive controlled release system was constructed by Liang *et al.* where the dual-responsive poly(*N*-isopropylacrylamide) (PNiPAM)-co-acrylic acid (AA) hydrogel core was enclosed in a silica shell via the self-assembly approach (PNiPAM/AA@SiO_2_ core–shell), which could respond to the inherent pH differences between cancers and normal tissues [99]. Drugs were loaded into the PNiPAM/AA@SiO_2_ nanoparticles under a lower temperature. With the increase of the acidity, the loaded drugs were rapidly released. Core-shell particles have a more uniform size and high dispersity, which is helpful to improve the release efficiency in cells compared with the common MSNs. These advantages indicate that such a system has great potential for selective release in tumor therapy applications. Another dual-responsive controlled release system was designed by Zhang and coworkers with thermo/pH-coupling sensitive polymer poly[(*N*-isopropylacrylamide)-co-(methacrylic acid)], which was grafted onto MSNs as a “valve” to regulate the diffusion of the embedded drugs in and out of the nanopores channels [100]. Under low temperature and high pH value conditions, the system showed a lower level of drug leakage, whereas it performed a higher level of drug release at higher temperature and lower pH value conditions, exhibiting an apparent thermo/pH controlled release system. Most crucially, the composite could specifically target Hep2 as a consequence of the covalent binding of folic acid molecules to it, laryngeal squamous cancer cells with folic acid receptors. Such a system showed a promising application in laryngeal carcinoma therapy.

#### 3.7.5. pH- and Enzyme-Responsive Controlled Release Systems

In 2014, a dual-responsive polymer-MSN system with the function of the “AND” logic gate was described by Gooding *et al*. [101]. Polycaprolactone (esterase degradable) was immobilized into the core of MSNs, while pH-responsive polyacrylic acid (PAA) was covered on the outer surface of the MSNs to build a PAA-PCL-MSN structure. The drug release behavior could only be observed in the presence of both low pH and esterase, which could be designed into an “AND” logic gate capable of selectively releasing drug molecules. In the same year, Xia and coworkers reported a novel dual-responsive nano-switch with natural chitosan end-capped MSNs to control the release of drugs [102]. The chitosan nano-switch strongly closed the nanopores of the MSNs to inhibit drug leakage under physiological conditions, but responded to lysozyme and acidic media to release the entrapped drug.

#### 3.7.6. pH- and Electrical-Responsive Controlled Release Systems

In 2014, Shi *et al*. evaluated a pH- and electrical-responsive controlled release system, which was composed of chitosan hydrogel with embedded MSNs [103]. The loaded particles, ibuprofen (IB)-MSNs, were dispersed in chitosan solution and then the complex IB-MSNs/chitosan film of 2 mm thickness was deposited as a hydrogel on the titanium electrode. The release of IB followed a near-zero-order profile when the hydrogel was deposited as a thick film on the titanium plate in buffers with different pHs, though its kinetics varied. Moreover, the release of IB from the IB-MSNs/chitosan complex system was triggered by negative potentials of the titanium electrode. The pH and electrical dual-responsive system that made chitosan/MSN hydrogel has a great prospect for controlled release applications.

#### 3.7.7. Light- and Redox-Responsive Controlled Release Systems

In 2009, Feng *et al*. developed a multi-responsive controlled release system by using a polymeric network as a capping agent with the versatile assembly/disassembly of CD-based supramolecular complex [104]. When a diazo-linker and a water-soluble ditopic guest molecule with an azobenzene group at each end have been added in the system, the polymer around the MSNs could be cross-linked to constitute a polymeric network, which was capable of blocking MSNs nanopores. Such cross-linking was possible because of the high binding affinity between the *trans*-azobenzene group and β-CD. Due to the azobenzene group and the disulfide bond on the diazo-linker, such a polymeric network responded to three stimuli. Firstly, the binding complex composed of the *trans*-azobenzene group and β-CD was dissociated when *trans*-azobenzene was transformed to *cis*-azobenzene upon UV irradiation. Therefore, the polymeric network could be opened. Secondly, the dissociation of the supramolecular complex and the release of the entrapped drug could also be achieved by the addition of competitive host α-CD, which has a much better affinity with the azobenzene group than β-CD. Furthermore, since β-CD was linked to the polymer main chains through the S-S bond, the polymeric network could also be opened by cleaving the disulfide bond in the presence of disulfide-reducing agents such as DTT.

#### 3.7.8. Light- and Temperature-Responsive Controlled Release Systems

**Figure 11:**
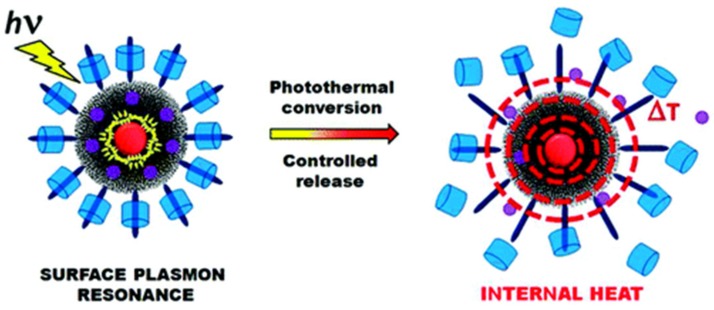
Schematic illustration of a multi-responsive Au@MSN@Valve. Reproduced with permission from [105]. Copyright American Chemical Society, 2012.

In 2012, Zink *et al*. discovered a facile method for one-pot synthesis of gold nanoparticles embedded in the MSNs (Au@MSNs), consisting of 20 nm gold cores inside 150 nm MSNs [105]. The nanovalves consist of cucurbit[6]uril rings encircling stalks that were attached to the ~2 nm nanopores. Plasmonic heating of the gold core could raise the local temperature and decrease the ring-stalk binding constant, thereby unblocking the nanopores and releasing the drug, as shown in Figure 11. In the same year, Zhao *et al*. utilized the ring structure of α-cyclodextrin and the azobenzene neck to achieve a light- and temperature-responsive controlled release system [106].

#### 3.7.9. Magnet- and Temperature-Responsive Controlled Release Systems

**Figure 12:**
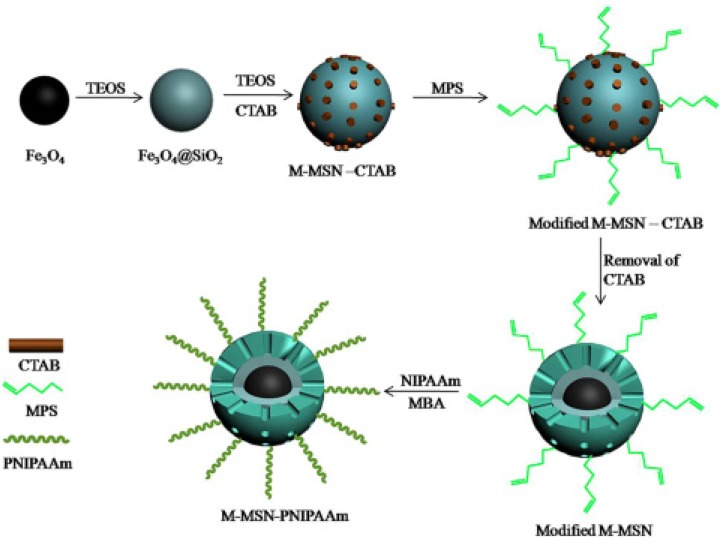
Schematic for the preparation process of M-MSN–PNIPAAm. Reproduced with permission from [108]. Copyright John Wiley and Sons, 2014.

In 2012, Vallet-Regí *et al*. reported a controlled release system where Fe_3_O_4_ nanocrystals were encapsulated inside the MSNs, and the MSNs’ surface was decorated with a thermoresponsive copolymer of poly(ethyleneimine)-*b*-poly(*N*-isopropylacrylamide) (PEI/NIPAM), forming a temperature-responsive controlled release system [107]. Moreover, superparamagnetic Fe_3_O_4_ nanocrystals were capable of providing the heating capability under alternate magnetic fields, which was also suitable for the hyperthermia treatment of cancer. In this work, the presented smart controlled release system was able to respond to external stimuli, temperature, or alternating magnetic field, subsequently releasing two different drugs, proteins, and small molecules. In 2014, a temperature and magnetic dual-responsive MSN-based controlled release system was developed by Chen and coworkers with magnetic (Fe_3_O_4_) nanoparticles as the core (M-MSN-PNIPAAm), as shown in Figure 12 [108]. The PNIPAAm layer on the surface of the MSNs triggered by various temperatures could be reversibly opened and closed, and this could regulate the uptake and release of drugs from M-MSN-PNIPAAm. M-MSN-PNIPAAm possessed superparamagnetic properties and could be guided to the target site by external magnetic fields. Therefore, M-MSN-PNIPAAm possessed functions of the dual-responsive and targeted release of drugs, showing significant potential application for controlled release systems.

### 3.8. Free-Blockage Controlled Release Systems

In 2014, our group reported a light-responsive controlled release system, which was achieved by adjusting the wetting of the MSNs’ surface [109]. At the starting stage, spiropyran and fluorinated silane (MSNs-FSP) molecules were modified onto the surface of MSNs to protect the MSNs from being wetted by water, successfully inhibiting the leakage of drugs. The conformational conversion of spiropyran from a “closed” state to an “open” state with 365 nm UV light irradiation caused the wetting of the surface and led to the release of drugs from the nanopores. In 2015, our group further designed a pH-responsive free-blockage controlled release system through controlling the hydrophobic/hydrophilic conversion of MSN nanopores [110], due to the deprotonation and protonation of the phenylamine (Ph) group, which possessed a convertible hydrophobic/hydrophilic property. The Ph groups attached onto the internal surface of the nanopores on the MSNs were hydrophobic enough to inhibit water entering into the nanopores, so as to entrap the drugs in the MSNs at pH 7.0. With a decrease of the pH value of the solution, the nanopores changed gradually to hydrophilic and allowed the intrusion of water and released the drugs due to the protonation of the Ph groups. This kind of release system greatly improved the efficiency of drug delivery in tumor tissues and reduced the side effects, as well as opening up a new prospect for constructing more practical controlled drug release systems.

### 3.9. MSN-Based Multifunctional Controlled Release Systems

Along with the deepening of study on MSN-based controlled release systems, scientists also introduced other materials into MSNs to build composite mesoporous silica nanostructures, and they also prepared a series of multifunctional controlled release systems which combined the targeting transport, chemotherapy, photothermotherapy, and imaging into one system.

In 2012, Qu *et al*. developed a novel near-infrared light-responsive controlled release system based on gold nanorods incorporated within MSNs that was surface-functionalized with aptamer DNA [111]. For the first time, the aptamer AS1411 was used as both the capping and targeting agent. Interestingly, when anticancer aptamer AS1411 formed a stable G-quadruplex structure, it showed high binding affinity to nucleolin, which is overexpressed in tumor cells, indicating that nucleolin on the cancer cell surface was a receptor for AS1411. The dehybrization of the linkage DNA duplex that anchored the G-quadruplex DNA on the surface of the MSNs was caused by the application of NIR light. The photothermal effect of the gold nanorods led to a fast rise in the local temperature, allowing the release of the entrapped drugs. This multifunctional platform integrated chemotherapy, photothermotherapy, and imaging into one system. Using a similar method, Chen and coworkers utilized gold nanorods/mesoporous silica core-shell structures to load doxorubicin, and successfully integrated thermal therapy and chemotherapy into one system for cancer treatment [112]. In 2013, a multifunctional boron neutron capture therapy (BNCT) agent based on MSNs was reported by Lin *et al*. The fluorescent dyes and trivalent galactosyl ligands were conjugated with surface-amino functionalization MSNs for imaging and cell targeting, respectively. And a great deal of *o*-carborane was stored in the hydrophobic nanopores [113]. This system exhibited a higher delivery efficiency of boron atoms, and a better effect of BNCT than commonly used method. The results demonstrated that the design of multifunctional MSNs was promising as potential BNCT agents for clinical application. In 2015, Liu and coworkers evaluated the multifunctional PEG-modified DOX-loaded MSN@CuS nanohybrids for the combination of photothermal therapy and chemotherapy, with a triggered release feature to hepatocellular carcinoma treatment [114]. The CuS nanoparticles coated on the surface of MSNs could serve as efficient photothermal therapy agents. Upon NIR irradiation, it could easily trigger the release of loaded drugs. The combination of thermal therapy and chemotherapy could further enhance the cancer-therapeutic effect compared with the single-therapeutic approach. In recent years, gadolinium oxide fluorescent nanoparticles [115], manganese oxide nanoparticles [116], and palladium silver alloy nanoparticles [117] were coated on the surface of MSNs for the design of a multifunctional system. Specifically, a series of nanoparticle-based multifunctional systems that exploited the core-shell structure of oxide magnetic nanoparticles/MSNs [118,119,120,121,122] was constructed with the functions of magnetic resonance imaging, directional transportation, and controlled release.

To sum up the above arguments, multifunctional controlled release systems are all constructed by adding some functional nanoparticles, or modifying some functional molecules, onto the MSNs’ surface, which would make these multifunctional controlled release systems more accommodative when encountering complex environments within the human body. These advantages make multifunctional controlled release systems a hot point in the future development of MSN-based controlled release systems.

## 4. Conclusions

In this review, we initially discussed the synthesis methods, the various functionalization approaches of MSNs, and the latest advances in controlled drug release systems. Some of the highlights of this review are the smart switches of the MSN-based controlled release system. The combination of cancer therapies and controlled release systems is shortly discussed in this review paper. Finally, we pay attention to the recent advancements in MSNs-based multifunctional controlled release systems.

In the past few years, MSN-based controlled release systems have been a hot spot due to their excellent properties and, therefore, a great number of smart designs have been reported, as mentioned above. Chemical stimuli-responsive controlled release systems are designed based on environment changes, which can only release the drug in the specific site and corresponding condition (such as redox, pH, enzyme), and that limits the development of system operation applications. In order to improve the operability of the controlled release system, physical stimuli-responsive controlled release systems have been designed (such as light, magnet, temperature). A comprehensive table summarizing the controlled release systems was briefly described, as shown in Table 1. Until now, the study of MSN-based controlled release systems has made significant advancements, but there is still a certain distance for clinical applications. For instance, it was found that the release behavior of drug-loaded MSNs is simple, and cannot achieve sequential release of multiple drugs; Vallet-Regí, Lin and Zink, and other research groups confirmed that MSNs have unique biocompatibility features, and cause no physical damage to cells or tissues, but the impact of MSNs and functionalized MSN materials’ enrichment and cycle metabolism *in vivo* on the normal physiology of the organism or cells is still inconclusive. Accurate and efficient controlled drug release behavior of MSN-based systems remains to be further expanded and studied in depth. In the future, controlled release system applications for organisms is the main direction. Multiple-responsive and multifunctional controlled release systems are where our hopes are pinned to solve this set of problems.

**Table 1:** Summary of functionalized MSN-based controlled release.

Category	Mechanism of Action	Components	Main Characteristics	Reference
Redox	disulfide linkages cleaved by oxidation-reduction reaction	CdS, Fe_3_O_4_, Au nanoparticles (AuNPs)	(1) easier to design and operate (2) the change of pH and redox homeostasis are internal signals of many serious diseases in human body	[21,22,23,24,25,26,27,28,29,30,31,32,33]
		polymer		
	different affinity between oxidized and reduced	pseudorotaxane		
pH	protonation	amine group contained compounds		[35,36,37,38,39,40,41,42,43,44,45,46,47,48,49,50,51,52,53,54,55,56,57]
		pseudorotaxane		
	acid hydrolysis	ZnO quantum dots		
		acetal group		
	oppositely charged ionic interaction	the negative group and the positive group		
Light	photodimerization	coumarin	remote responsiveness, non-invasiveness, highly controllable, low toxicity, convenient operation	[16,57,58,59,60,61,62,63,64,65]
	photocleavage	cyclobutane dimer		
	photoisomerization	azobenzene, spiropyrane		
Enzyme	catalyze the hydrolysis of complex	biotin-avidin complex	better biocompatible, specificity, accurate responsive	[75]

To address these issues, the future research work should be focused on: (1) the design of responsive switches with higher sensitivity, stronger anti-interference, and more accuracy and reliability, as well as multi-responsive and logic-responsive switches to apply to various complicated conditions; (2) the research of multifunctional systems to achieve unity of recognition, diagnosis, and treatment; (3) to improve the efficiency of cellular uptake of controlled release systems, to further optimize the MSNs’ biocompatibility, and to improve the MSNs’ stability in cells and in intracellular release and therapeutic function; (4) continuous study of the MSN-based controlled release system to apply to developing clinical research. The MSN-based controlled release system has achieved many important advances. Scientists have not only built a powerful group of external stimuli-responsive nano-switches, but they have also gradually increased the biocompatibility, degradability, and targeted release of MSNs, as well as multiple-responsive controlled release features. Particularly, the controlled release systems based on DNA and other biological molecules are established due to their high degree of biocompatibility, their precise controlled release capability, their use in milder conditions for controlled release and other advantages, and in their ability to lay a solid foundation for the clinical application of MSN-based controlled release systems in the human body. With further research, MSN-based controlled release systems can be widely used in environmental protection and management, energy conservation, plant pest control, corrosion and inhibition on the surface of material, and other fields.
